# Telomere length analysis in monoclonal B-cell lymphocytosis and chronic lymphocytic leukemia Binet A

**DOI:** 10.1590/1414-431X20176019

**Published:** 2017-04-13

**Authors:** F.M. Furtado, P.S. Scheucher, B.A. Santana, N.F. Scatena, R.T. Calado, E.M. Rego, D.M. Matos, R.P. Falcão

**Affiliations:** 1 Divisão de Hematologia, Departamento de Clínica Médica, Faculdade de Medicina de Ribeirão Preto, Universidade de São Paulo, Ribeirão Preto, SP, Brasil; 2 Hospital Universitário Walter Cantidio, Faculdade de Medicina de Fortaleza, Universidade Federal do Ceará, Fortaleza, CE, Brasil

**Keywords:** Chronic lymphocytic leukemia, Monoclonal B-cell lymphocytosis, Telomere length

## Abstract

Monoclonal B-cell lymphocytosis (MBL) is an asymptomatic clinical entity characterized by the proliferation of monoclonal B cells not meeting the diagnosis criteria for chronic lymphocytic leukemia (CLL). MBL may precede the development of CLL, but the molecular mechanisms responsible for disease progression and evolution are not completely known. Telomeres are usually short in CLL and their attrition may contribute to disease evolution. Here, we determined the telomere lengths of CD5^+^CD19^+^ cells in MBL, CLL, and healthy volunteers. Twenty-one CLL patients, 11 subjects with high-count MBL, and 6 with low-count MBL were enrolled. Two hundred and sixty-one healthy volunteers aged 0 to 88 years were studied as controls. After diagnosis confirmation, a flow cytometry CD19^+^CD5^+^-based cell sorting was performed for the study groups. Telomere length was determined by qPCR. Telomere length was similar in the 3 study groups but shorter in these groups compared to normal age-matched subjects that had been enrolled in a previous study from our group. These findings suggest that telomere shortening is an early event in CLL leukemogenesis.

## Introduction

Chronic lymphocytic leukemia (CLL) is the most common leukemia of the western world, with an annual incidence of 5.1 cases/100,000 persons. Over the last years, advances in multi-parameter flow cytometry have provided the identification of a small population of monoclonal B lymphocytes with identical CLL immunophenotype. This condition is found in 0.6 to 12% of adults with normal blood cell counts (1) being called monoclonal B-cell lymphocytosis (MBL) (2).

MBL may be classified as high-count (HC) and low-count (LC) (3). HC MBL is usually diagnosed in asymptomatic subjects with mild lymphocytosis and LC MBL occurs in asymptomatic subjects with normal blood counts that have been submitted to flow cytometry screening (4).

As occurs in CLL, MBL is more frequent in men, especially in relatives of CLL patients (5,6) and its frequency increases with age (5,7).

Telomeres provide protection against threats to the genome and are reduced in every cell cycle due to the inability of the DNA polymerase to replicate the chromosome's 3′ ends (8). Telomeric erosions may interfere with telomeres function to protect the chromosomes, causing genetic instability (9) and may have an important role in common human tumors, like CLL (9,10).

Studies addressing the molecular and genetic basis of MBL may help to elucidate initial points of CLL pathophysiology and, therefore, increase knowledge about the origin of CLL. As far as we know, telomere length (TL) has never been previously investigated in subjects with the diagnosis of MBL.

Here, we hypothesized that telomere shortening may be present in MBL and may have a role in the initial monoclonal B-cell expansion.

## Material and Methods

### Cell samples from normal individuals, individuals with LC MBL, HC MBL, and patients with CLL

We analyzed samples from 6 individuals with LC MBL, 11 with HC MBL and 21 patients with CLL Binet A who were followed at Hospital das Clínicas, Faculdade de Medicina de Ribeirão Preto, SP, Brazil. The control group was obtained from data of 261 healthy volunteers aged 0 to 88 years previously enrolled in another study from our group (11). The study was reviewed and approved by the Institution’s Research Ethics Review Board and written informed consent was obtained from all participants in accordance with the Declaration of Helsinki.

Using cell sorting flow cytometry, CD19^+^CD5^+^ lymphocytes were purified from peripheral blood of individuals from the three groups. Post-preparation purity, as assessed by flow cytometry (available on 37/48 volunteers), indicated that the isolated cells were predominantly (median of 86%) the desired cells.

### DNA isolation and TL analysis

DNA was extracted from the isolated CD5^+^ B lymphocytes of the 3 study groups with the Gentra Puregene Blood¯ kit (Qiagen, Netherland), according to the manufacturer's protocol. TL was determined by real time polymerase chain reaction (PCR), as previously described (12) and data are reported as telomere/single copy gene (T/S) ratio. Briefly, two separate PCR runs were performed for each sample, the first to determine the cycle threshold (Ct) value for telomere amplification, and the second to determine the Ct value for control gene amplification. A standard curve was generated in each run, consisting of reference DNA diluted serially. Both reference and sample DNA were analyzed in triplicate (16 ng DNA/aliquot).

The Ct data generated in both runs were used to calculate relative T/S values for each sample: T/S = 2^-ΔΔCt^. CV less than 2% was accepted for telomere reactions and less than 1% for single gene reactions. All samples were studied in triplicate.

Using these criteria, 5 individuals were excluded, 2 from the LC MBL group, 2 from the HC MBL group, and 1 from the CLL group.

### Statistical analysis

TL was analyzed by two different methods. First, data from all the control group volunteers were analyzed (TL related to age) and the covariance test (ANCOVA) was performed to compare the four groups. To discard possible age-related bias, another analysis (age-matched and adjusted TL) was done using data from 43 controls aged 50 years or older (medium 58; range 50-88) and non-parametric Kruskal-Wallis and confirmatory Dunn test were used. All tests were considered to be statistically significant at the P<0.05 level.

## Results

Characteristics of participants are shown in Table 1. Median TL was 0.32 (range, 0.13 to 0.78), 0.21 (range, 0.13 to 0.48) and 0.42 kb (range, 0.36 to 0.45) for CLL, HC MBL and LC MBL, respectively.

**Table 1:**
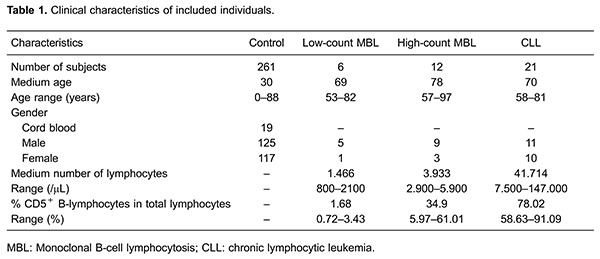
Clinical characteristics of included individuals.

**Figure 1:**
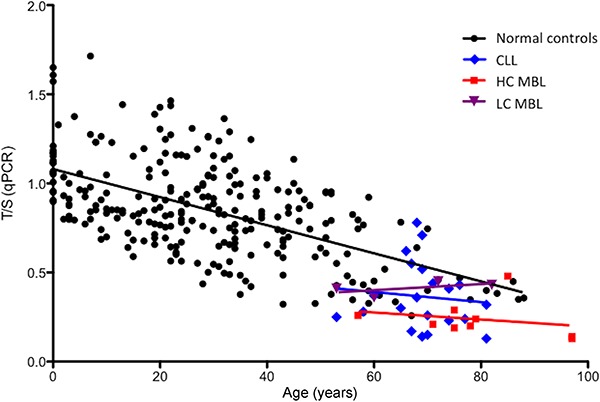
Telomere length in relation to age in normal controls, low-count monoclonal B-cell lymphocytosis (population screening MBL), high-count MBL (clinical MBL) and chronic lymphocytic leukemia (CLL) patients measured in telomere/single copy gene ratio (T/S).

TL related to age was similar among the three groups. When compared to healthy controls, telomeres from individuals with abnormal B-cell phenotype were significantly smaller than telomeres from normal subjects (CLL and HC MBL, P<0.001; LC MBL, P=0.007). In healthy individuals, TL from peripheral blood leukocytes shortened with aging, but in the three patient groups analyzed, clonal B-cells TLs were equally short regardless of the patient's age (Figure 1).

**Figure 2:**
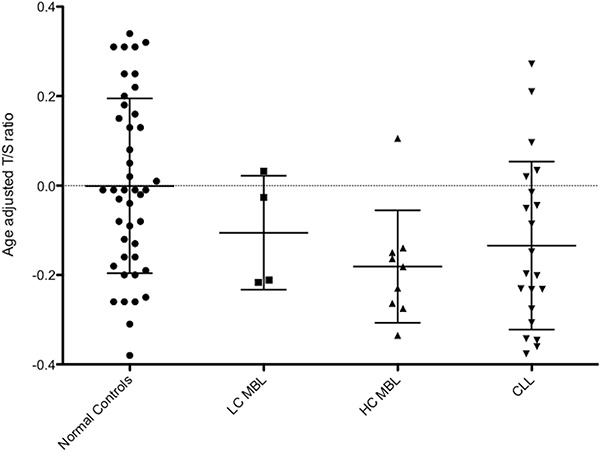
Peripheral blood leukocyte and clonal B-cell telomere length matched for age and adjusted in healthy controls, low-count monoclonal B-cell lymphocytosis (LC MBL), high-count MBL (HC MBL) and Binet A chronic lymphocytic leukemia (CLL) patients. Horizontal lines indicate median and interquartile range. T/S: telomere/single copy gene.

Age-matched and adjusted TL was shorter in CLL and HC MBL compared to healthy controls (P<0.05; Figure 2). TL also tended to be shorter in LC MBL compared to healthy subjects, although not reaching statistical significance probably due to the low number of individuals in this group (Figure 2).

## Discussion

The finding that average telomere length is shorter in CLL than in normal controls has been demonstrated previously by various authors using different methodologies (13–15). This has been associated to unmutated IGHV status (15) and to worse prognosis (16). Our finding confirms these previous reports.

For the first time, our group compared the TL from HC and LC MBL, CLL Binet A and normal controls. We found the 3 study groups to have similar TL and shorter than the general population's. However, age-matched and adjusted TL was similar in LC MBL and controls. These findings must be confirmed by further investigations, since our LC MBL group was too small to allow definite conclusions.

Significant similarities have been identified regarding the frequencies of IGHV genes between HC MBL and initial stages CLL, although findings from LC MBL were different from aforementioned groups (17). However, other authors also found some biologic similarities between these 3 entities. Not only HC MBLs but also LC MBLs bear cytogenetic abnormalities common in CLL, including 13q-, 17p- and trisomy 12 (1,18).

Notwithstanding the similarities between HC MBL and initial stage CLL with regard to cytogenetic abnormalities (1) and IGHV genes (17) – and the dissimilarities of these two entities when compared to LC MBL found in some previous studies – our finding suggests that the absence of difference in TL among LC MBL, HC MBL and CLL Binet A supports the hypothesis that inside the "MBL" label there may be a combination of non-progressive and potentially progressive entities. Moreover, the presence of short telomeres already inside the small abnormal B-cell clone of HC MBL cases, compared to the general population, suggested that it may be part of the initial events in CLL physiopathology. Finally, our findings were in accordance with most recent evidence suggesting that the primary leukemogenic event occurs very early in CLL, probably involving multipotent, self-renewing hematopoietic stem-cells (19). This makes the natural history of CLL comparable with that of other tumors, where a pre-malignant lesion (here, MBL) progresses toward a full malignant disease (20).

In conclusion, we showed that TL was similar among HC MBL and CLL Binet A, which suggests that telomere erosion may be an early event in CLL biology and points to a *continuum* from HC MBL to CLL.
